# Supplementary material for: HIVprotI: an integrated web based platform for prediction and design of HIV proteins inhibitors
Source: J Cheminform. 2018 Mar 9;10:12. doi: 10.1186/s13321-018-0266-y (PMC5845081; doi:10.1186/s13321-018-0266-y)
Supplement: Supplementary file 2 — Additional file 2. Source code of HIVProtI web server. [file 13321_2018_266_MOESM2_ESM.zip › HIVprotI_Source-code/hivprotI/weka-3-6-6/doc/weka/filters/UnsupervisedFilter.html]

UnsupervisedFilter


---


|  |  |  |
| --- | --- | --- |
| **Overview** \| **Package** \| **Class** \| **Tree** \| **Deprecated** \| **Index** \| **Help** \| **Weka's home** | |  |
| **PREV CLASS**   NEXT CLASS | **FRAMES**    **NO FRAMES**     **All Classes** |  |
| SUMMARY: NESTED \| FIELD \| CONSTR \| METHOD | DETAIL: FIELD \| CONSTR \| METHOD |  |


---


## weka.filters Interface UnsupervisedFilter

**All Known Implementing Classes:**: AbstractTimeSeries, Add, AddCluster, AddExpression, AddID, AddNoise, AddValues, Center, ChangeDateFormat, ClusterMembership, Copy, Discretize, FirstOrder, KernelFilter, MakeIndicator, MathExpression, MergeTwoValues, MultiInstanceToPropositional, NominalToBinary, NominalToString, NonSparseToSparse, Normalize, Normalize, NumericToBinary, NumericTransform, Obfuscate, PKIDiscretize, PrincipalComponents, PropositionalToMultiInstance, Randomize, RandomProjection, Remove, RemoveFolds, RemoveFrequentValues, RemoveMisclassified, RemovePercentage, RemoveRange, RemoveType, RemoveUseless, RemoveWithValues, Reorder, ReplaceMissingValues, Resample, ReservoirSample, SparseToNonSparse, Standardize, StringToNominal, StringToWordVector, SwapValues, TimeSeriesDelta, TimeSeriesTranslate

---

``` public interface UnsupervisedFilter ```

Interface for filters that do not need a class attribute.

**Version:**
:   $Revision: 1.2 $

**Author:**
:   Richard Kirkby (rkirkby@cs.waikato.ac.nz)

---

---


|  |  |  |
| --- | --- | --- |
| **Overview** \| **Package** \| **Class** \| **Tree** \| **Deprecated** \| **Index** \| **Help** \| **Weka's home** | |  |
| **PREV CLASS**   NEXT CLASS | **FRAMES**    **NO FRAMES**     **All Classes** |  |
| SUMMARY: NESTED \| FIELD \| CONSTR \| METHOD | DETAIL: FIELD \| CONSTR \| METHOD |  |


---
